# Effects of Fallow Management Practices on Soil Water, Crop Yield and Water Use Efficiency in Winter Wheat Monoculture System: A Meta-Analysis

**DOI:** 10.3389/fpls.2022.825309

**Published:** 2022-04-27

**Authors:** Muhammad Adil, Shaohong Zhang, Jun Wang, Adnan Noor Shah, Mohsin Tanveer, Sajid Fiaz

**Affiliations:** ^1^Shaanxi Key Laboratory of Earth Surface System and Environmental Carrying Capacity, College of Urban and Environmental Science, Northwest University, Xi’an, China; ^2^State Key Laboratory of Soil Erosion and Dryland Farming on the Loess Plateau, Institute of Soil and Water Conservation, Chinese Academy of Sciences and Ministry of Water Resources, Yangling, China; ^3^Department of Agricultural Engineering, Khwaja Fareed University of Engineering and Information Technology, Rahim Yar Khan, Pakistan; ^4^Tasmanian Institute of Agriculture, University of Tasmania, Hobart, TAS, Australia; ^5^Department of Plant Breeding and Genetics, The University of Haripur, Haripur, Pakistan

**Keywords:** wheat mono-cropping system, fallow, tillage, mulching, soil water conservation

## Abstract

Winter wheat monoculture is a predominant cropping system for agricultural production in dry areas. However, fallow management effects on soil water conservation and crop yield and water use have been inconsistent among studies. We selected 137 studies and performed a meta-analysis to test the effects of tillage and mulching during the fallow period on precipitation storage efficiency (PSE), soil water storage at wheat planting (SWSp), crop yield, evapotranspiration (ET), and water use efficiency (WUE). Compared to conventional tillage (CT), conservation tillage during fallow period overall increased PSE, SWSp and wheat yield by 31.0, 6.4, and 7.9%, respectively, but did not affect ET and WUE. No tillage (NT) had a better performance on soil water conservation during fallow period but a similar effect on wheat yield and WUE compared to reduced tillage (RT) and subsoil tillage (ST). Compared to no mulching, fallow mulching practices overall increased PSE by 19.4%, but had a non-significant impact on SWSp, wheat yield, and ET. Compared to straw mulching, film mulching, and stubble mulching during fallow period, cover cropping as a biological mulching decreased SWSp, wheat yield, and WUE significantly. Wheat WUE was improved by straw mulching but not affected by film mulching and stubble mulching. Strong interactions between tillage method and mulching practices were found for most variables. NT with fallow mulching or with no mulching exhibited a greater impact on soil water conservation during fallow period compared to other combinations. The effects of tillage and mulching during fallow period on soil water conservation and wheat yield and water use also varied with soil and climatic conditions. Overall, NT in combination with straw mulching significantly increased SWSp, PSE, wheat yield, and WUE and can be the best fallow management practice for winter wheat production in varying edaphic and climatic conditions.

## Introduction

Wheat (*Triticum aestivum* L.) is one of the most important cereal crops throughout the world (Bruning et al., 2020; Bukhari et al., 2021). It is planted on more than 2.2 million acres in the inland Pacific Northwest of the USA alone (Schillinger and Papendick, 2008) and an additional 4.3 million hectares in the Chinese Loess Plateau, providing 40% of the food grains in China (Tong et al., 2003). In the arid and semiarid regions where water and heat resources are limited, wheat is generally cultivated in mono-culture following with a short or long fallow period. A short 3-month summer fallow between the harvest in late June and planting in late September of winter wheat, is adopted on the Loess Plateau of China (Shangguan et al., 2002; Wang et al., 2011) while a long fallow of more than 14 months, growing one crop in two years, is generally practiced in the western United States (Smiley and Uddin, 1993; Peterson et al., 1996; Tanaka and Anderson, 1997; Nielsen and Vigil, 2010). Improving precipitation storage efficiency (PSE) during the fallow period could significantly increase wheat yield and profitability of the winter wheat mono-culture system in dryland areas.

After harvesting winter wheat at the beginning of the rainy season in the drylands, local farmers generally plow the soil to increase soil moisture retention through dust-mulch effects (Shah et al., 2017). Although such conventional tillage (CT) can increase water permeability temporarily, but it could increase water loss due to evaporation compared to undisturbed soil (Jin et al., 2007). Compared to CT, conservation tillage practices including subsoil tillage (ST), reduced tillage (RT), and no tillage (NT) have been widely adopted to save water during the fallow period (Rasmussen, 1999; Schillinger, 2001; Lampurlanes et al., 2002; Wang et al., 2003; Li et al., 2007; He et al., 2009). Changes in soil water-related properties acquired with NT depend on several factors, including initial soil properties, land management history, weather conditions, and type and tillage intensity (Mahboubi et al., 1993). RT improved soil infiltration, reduced surface runoff and evaporation, and ultimately increased soil water content (Zhai et al., 1990). According to Patrignani et al. (2012), the effect of conservation tillage on soil water recharge during the fallow period varied with climatic and soil conditions (Mahboubi et al., 1993). Conservation tillage increased PSE with a 27% higher value compared to CT in Ohio and 25% higher in Colorado, USA (Farahani et al., 1998). Similarly, NT and ST increased SWSp by 10.2 and 11.5%, respectively, in northwestern China (Hou et al., 2012). The PSE increased by 81% and soil water storage at planting (SWSp) by 188 mm under NT than CT in the Great Plains (Nielsen and Vigil, 2010), and the PSE increased by 43% at North Platte (Smika and Wicks, 1968), and by 38% at Sidney, MT (Tanaka and Aase, 1987).

In dryland agriculture, soil surface cover management determines soil moisture loss, water storage, and crop productivity. Covering the soil surface with plastic film has become a broadly used technique to improve crop productivity in arid regions of China (Xie et al., 2005), including covering all or part of the soil surface (ridge mulching and planting in furrows) during the whole year or part of the growing period (Li et al., 1999). Wang et al. (2018) reported that straw mulching increased PSE by 13–16% compared to no mulching during summer fallow. Straw mulching significantly increased soil water content and wheat yield by 23% each and water use efficiency (WUE) by 33% (Zhang et al., 2015). Similarly, straw mulching enhanced winter wheat grain yield and WUE by 13–25% compared to no mulching (Chakraborty et al., 2010). Film mulching during summer fallow increased SWSp by 13% (He et al., 2016) and 56% (Ren et al., 2019), making it the highest soil-water storage (50 mm) in winter wheat cropping system. Wheat grain yield was significantly lower with cover cropping than bare fallow in the first year of study (Zhang et al., 2015). However, more information is needed to understand whether different mulching and tillage methods can always increase water storage and crop yields under different soil types and climatic conditions.

A meta-analysis is a valuable tool that uses the effect size of individual studies combining the data from different management practices originating from different soil and climate conditions (Hedges et al., 1999; Hungate et al., 2009). In this study, we collected the data available in the literature and conducted a meta-analysis to check the overall and individual effect of conservation tillage and mulching methods on soil and plant parameters compared to conventional tillage and bare fallow cropping patterns under different edaphic and climate conditions. We hypothesized that conservation tillage and mulching would improve PSE, SWSp, winter wheat yield, and WUE compared to conventional tillage and bare fallow. Relative effects on soil water parameters may differ within tillage practices, mulching methods, and edaphic and climatic conditions.

## Materials and Methods

### Data Collection

Peer-reviewed journal articles published between 1968 and 2021 were searched in Google Scholar^1^ and Web of Science^2^ to evaluate the effects of fallow tillage and mulching methods on soil water and crop yield and water use in mono-culture winter wheat system. We used soil water, precipitation storage efficiency, wheat yield, and WUE along with conventional tillage, conservation tillage, bare fallow, and mulching as keywords. Initially, about 1,326 publications were collected and then screened using criteria as follows:

• Tillage and/or mulching studies should be carried out in the mono-culture winter wheat cropping system experiments in a field.
• Each study should have a control treatment, e.g., conventional tillage (for tillage comparison) and bare fallow (for mulching comparison) with similar edaphic and climatic conditions.
• SWSp, PSE, yield, ET, and WUE should be compared between conservation tillage methods (NT, RT, and ST) and CT or between straw mulching, plastic film mulching, cover cropping (as a biological mulching), stubble mulching, and a bare fallow without mulching. All crop residues should be removed immediately at harvest of the main crop, and the land kept bare during the whole fallow period as a control treatment for mulching.
• Experiments should be conducted in a rain-fed system with no irrigation applied during the entire experimental period. The simulation model and multi-cropping studies were excluded.

**FIGURE 1:**
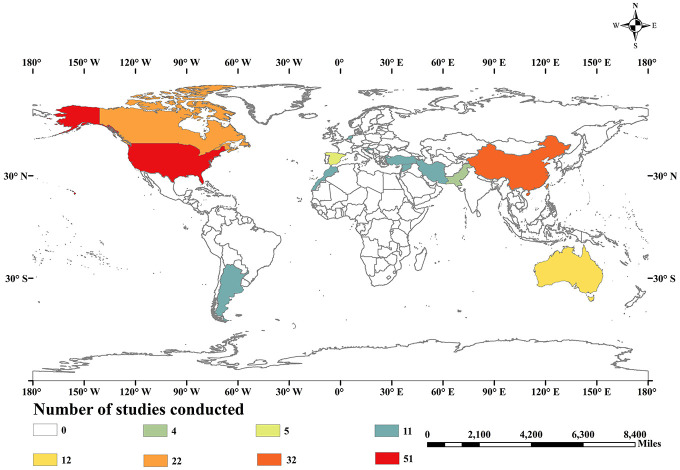
Distribution of 137 experimental sites around the globe from where the data was collected for the meta-analysis.

A brief description of tillage and mulching practices used in this meta-analysis is provided in Table 1, which further clarifies search criteria. Finally, 2,187 observations for tillage and 1,655 observations for mulching were collected from 137 studies from 76 sites, covering 15 countries (Figure 1). Group homogenization of the data was accomplished according to different tillage and mulching methods. Data from the figures were extracted by using GetData graph digitizer 2.20 software.^3^ By using standard error (*SE*) value, we calculated the standard deviation (*SD*) value by using the following formula:


(1) $$SD=SE\times \sqrt{n}$$

**TABLE 1:** Description of tillage and mulching practices used for fallow management in a wheat monoculture system.

Management practices	Brief description
**Tillage**	
Conventional tillage (CT)	The field is tilled using a tractor-mounted moldboard plow to a depth of 20–25 cm for 4–6 times during a fallow period (with or without mulching).
No-tillage (NT)	Land remained bare and undisturbed during the entire fallow period until the next planting, mostly no-till planter was used to plant the main crop.
Reduced tillage (RT)	Soil was inverted and plowed to a depth of 25–30 cm combined with generally harrowing at a depth of 5–8 cm in the primary tillage.
Subsoil tillage (ST)	Soil is plowed by a deep soil chisel to a depth of 30–35 cm, with its adjustable wings making the distance between two ends by at least 60 cm.
**Mulching**	
Bare fallow (control)	At winter wheat harvest, all the residual straw was removed, and the land was kept fallow until sowing of the next wheat crop (by keeping tillage constant).
Straw mulching	Wheat straw was evenly distributed over the soil surface at an average rate of 6 t ha^–1^ during the entire fallow period.
Film mulching	A plastic film of generally 0.008 mm thickness was used as mulching during the whole fallow period.
Cover cropping	Growing of cover crops during the entire fallow period.
Stubble mulching	All of the residual straw of wheat was kept in place during the entire fellow period.

Where “*n*” represents the number of samples.

In publications where PSE, ET, or WUE were not calculated, but the SWS and fallow precipitation were available, we manually calculated these variables using the following equations (Tanaka and Anderson, 1997; Nielsen and Vigil, 2010; Zhang et al., 2015).


(2) $$PSE=\frac{\Delta SWS}{P_{f}}$$

Where Δ*SWS* is the difference in SWSp and harvest during the fallow period, and *P*_*f*_ is the precipitation during the fallow period.

Wheat WUE was calculated as:


(3) $$WUE=\frac{Yield}{ET}$$

Where ET is evapotranspiration, whereas ET was determined by following soil water balance equation:


(4) $$ET=\Delta SWS+P_{g}$$

Where Δ*SWS* is the change in soil water storage during wheat growing season and *P*_*g*_ represents the precipitation during the growing season.

Mean annual precipitation (MAP), mean annual air temperature (MAT), along with soil texture of the experimental plots, were also recorded for each study. If the information about soil properties, and climatic conditions were not found in the study, then these observations were searched in an online search engine.^4^ Soil textures were categorized into three groups: fine (clay, clay loam, silty clay loam, and silty clay), medium (silt, loam, silt loam, and sandy silt loam) and coarse (sandy loam, sandy clay loam, loamy sand and sand) according to the USDA soil classification system. MAP was classified into <400 mm, 400–600 mm, and >600 mm, and MAT was grouped into frigid (<8°C), mesic (8–15°C), and thermic (>15°C) (Knorr et al., 2005).

### Data Analysis

The effects of conservation tillage compared to CT and of fallow mulching compared to no mulching were determined with the help of response ratio (RR) and the natural log of RR taken as effect size (Hedges et al., 1999):


(5) $$RR=Ln\left(\frac{X_{COT}}{X_{CT}}\right)=Ln\left(X_{COT}\right)-Ln\left(X_{CT}\right)$$

(6) $$RR=Ln\left(\frac{X_{NM}}{X_{M}}\right)=Ln\left(X_{NM}\right)-Ln\left(X_{M}\right)$$

Where *X*_*COT*_ and *X*_*CT*_ exhibits arithmetic mean fluxes of soil and plant parameters (SWSp, PSE, yield, ET, and WUE) with conservation tillage and CT, respectively. The comparison between CT and COT was determined separately for each experiment studied. *X*_*NM*_ and *X*_*M*_ exhibit arithmetic mean fluxes with no mulching and fallow mulching practices, respectively. For each experiment studied, the comparison between fallow mulching and no mulching was determined separately.

Error variance (*V*) within each experiment studied was calculated with the following formula (Hedges et al., 1999).


(7) $$V=\frac{S_{COT}^{2}}{N_{COT}X_{COT}^{2}}+\frac{S_{CT}^{2}}{N_{CT}X_{CT}^{2}}$$

(8) $$V=\frac{S_{M}^{2}}{N_{M}X_{M}^{2}}+\frac{S_{NM}^{2}}{N_{NM}X_{NM}^{2}}$$

Where *S*_*COT*_ and *S*_*CT*_ are SD values for conservation tillage and CT, *N*_*COT*_ and *N*_*CT*_ indicate number of replications for conservation tillage and CT, and *X*_*COT*_ and *X*_*CT*_ are mean for conservation tillage and CT, *S*_*M*_ and *S*_*NM*_ are SD values for fallow mulching and no mulching, *N*_*M*_ and *N*_*NM*_ are number of replications for fallow mulching and no mulching, and *X*_*M*_ and *X*_*NM*_ are water storage with no mulching and fallow mulching, respectively.

The reciprocal of the variance (*V*) taken as the weight (*W*) for each RR was determined by the following formula (Lucas et al., 2011):


(9) $$W=\frac{1}{V}$$

Studies with more variance are weighed less heavily during analysis than those with less variance, a method given by Hedges et al. (1999). Individual RR value of conventional and conservation tillage was used to calculate the overall mean response ratio (RR_*E*++_) as follows:


(10) $$RR_{E++}=\frac{\sum_{i=1}^{n}\sum_{j=1}^{m}W_{ij}RR_{ij}}{\sum_{i=1}^{n}\sum_{j=1}^{m}W_{ij}}$$

Within each category, “n” represents the number of treatments while “m” is the number of comparisons. The standard error of RR_E++_ was calculated as:


(11) $$SE\left(RR_{E++}\right)=\sqrt{\frac{1}{\sum_{i=1}^{n}\sum_{j=1}^{m}W_{ij}}}$$

In order to analyze the impact of conservation tillage methods on soil and crop parameters (SWSp, PSE, yield, ET, and WUE), random model MetaWin 2.1 (Sinaure Associate Inc., Sunderland, United Kingdom) was used to calculate the mean effect size of bias-based bootstrap at 95% confidence Interval. The impact of the conservation tillage methods was measured significant if the 95% confidence interval did not overlap with the zero line. Correlations of the RRs of wheat yield, ET, and WUE to that of SWSp were conducted using the Origin 2018 software (OriginLab Corporation, United States).

## Results

### Tillage Effect on Precipitation Storage Efficiency and Soil Water Storage at Wheat Planting

**FIGURE 2:**
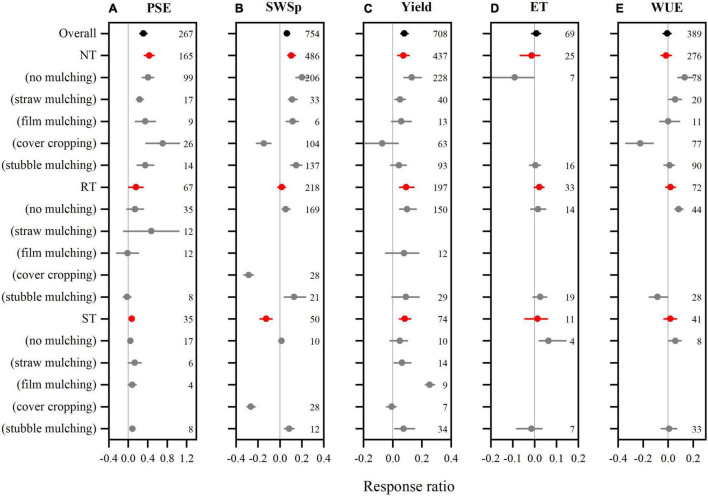
The mean response ratios of precipitation storage efficiency (PSE, **A**), soil water storage at wheat planting (SWSp, **B**), winter wheat grain yield **(C)**, evapotranspiration (ET, **D**), and water use efficiency (WUE, **E**) to conservation tillage methods during fallow period compared to conventional tillage (CT) and their interactions with fallow mulching practices. The horizontal line represents the bootstrapped 95% confidence interval. Conservation tillage methods are no tillage (NT), reduced tillage (RT), subsoil tillage (ST). The reference line (RR = 0) specifies no variation between conservation tillage and conventional tillage. Numbers accompanying the bootstrapped 95% confidence intervals designate the number of observations for comparisons.

**FIGURE 3:**
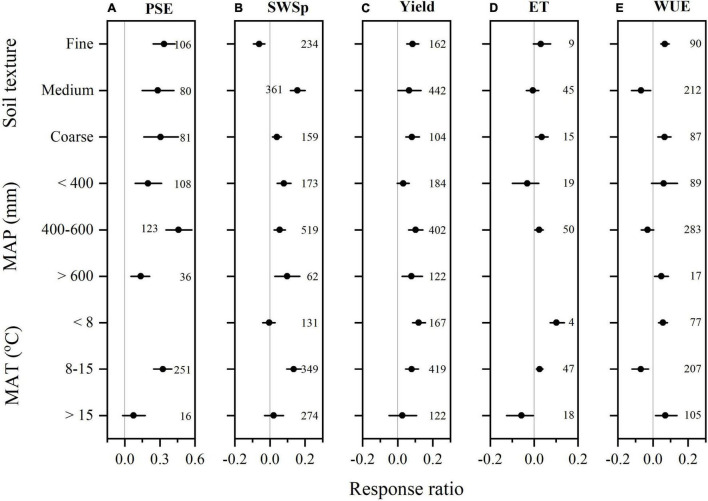
The mean response ratios of precipitation storage efficiency (PSE, **A**), soil water storage at wheat planting (SWSp, **B**), winter wheat grain yield **(C)**, evapotranspiration (ET, **D**), and water use efficiency (WUE, **E**) to conservation tillage methods during fallow period compared to conventional tillage and their bootstrapped 95% confidence intervals (Horizontal line) as affect by soil texture, mean annual precipitation (MAP), and mean annual air temperature (MAT). The reference line (RR = 0) specifies no variation between conservation tillage and conventional tillage. Numbers accompanying the bootstrapped 95% confidence intervals designate the number of observations for comparisons.

We collected 267 paired observations for PSE and 754 for SWSp (Figures 2A,B). Data exhibited high heterogeneities as indicated by high *Qt* values of 256 and 767 for PSE and SWSp, respectively. Conservation tillage methods overall increased PSE by 31.0% (*P* < 0.05) compared to CT (Figure 2A). Among conservation tillage methods, the highest increase in PSE was observed with NT (42.5%), followed by RT (15.2%) and ST (7.0%). The effect of PSE to NT also varied with mulching practices. The RR of PSE was greater with NT cover cropping than NT straw mulching. However, no significant differences among mulching practices in the RRs of PSE were found under RT and ST compared to CT. The RR of PSE to conservation tillage methods compared to CT did not vary with soil textures (Figure 3A). The enhancement of conservation tillage on PSE was greater when MAP was 400–600 mm than < 400 and > 600 mm. Conservation tillage also increased PSE in the regions when MAT was 8–15°C and had no significant effect when MAT was > 15°C.

Compare to CT, conservation tillage overall increased SWSp by 6.4% (*P* < 0.05, Figure 2B). However, such positive effect on SWSp varied with tillage methods. NT increased SWSp by 10.5%, but ST decreased by 12.5%. Strong interactions were found between tillage methods and mulching practices. All conservation tillage methods increased SWSp with mulching practices except for cover cropping. Both NT and RT decreased SWSp with cover cropping but increased with other mulching practices. The RR of SWSp for ST was positive with cover cropping but negative with stubble mulching. The RR of SWSp to conservation tillage to CT was negative in fine soils, but positive in medium and coarse soils (Figure 3B). The effect of Conservation tillage on SWSp did not vary with MAP, and was positive when MAT was 8–15°C but not significant when MAT was <8 and > 15°C.

### Tillage Effect on Winter Wheat Yield, Evapotranspiration, and Water Use Efficiency

Overall, 708 observations were measured for winter wheat yield, 69 for ET, and 389 for WUE. Data was heterogeneous for yield (*Qt* = 689) and WUE (*Qt* = 372), but not for ET (*Qt* = 70) (Figures 2C–E). Conservation tillage methods increased winter wheat yield by 7.9% compared to CT (*P* < 0.05, Figure 2C). The categorical meta-analysis showed that NT, RT, and ST increased winter wheat yield by 7.3, 9.2, and 8.3%, respectively. When combined with mulching practices, however, winter wheat yield was only enhanced by NT no mulching, NT straw mulching, RT no mulching, ST straw mulching, and ST film mulching. The RR of wheat yield to conservation tillage compared to CT was consistent with soil textures, MAP, and MAT, although conservation tillage had a non-significant impact on wheat yield when MAT was > 15°C (Figure 3C).

There was no significant effect of overall conservation tillage methods on winter wheat ET. However, NT no mulching decreased ET by 9.1% and ST no mulching increased by 6.3% (*P* < 0.05, Figure 2D). With all other management practices, ET remained non-significant. Conservation tillage increased ET in coarse soils, but its effect on ET was not significant in fine and medium soils (Figure 3D). The RR of ET to conservation tillage compared to CT was also positive when MAP was 400–600 mm, but non-significant when MAP was < 400 mm. Conservation tillage increased ET when MAT was < 15°C but and decreased it when MAT was > 15°C.

Winter wheat WUE was also not significant for conservation tillage during fallow period (Figure 2E). However, strong interactions with mulching practices were found for NT, RT, and ST. Compared to CT, NT increased wheat WUE by 13.3%, and 5.6% with no mulching and straw mulching, respectively, but decreased by 22.4% with cover cropping. Similarly, RT increased wheat WUE by 8.4% with no mulching but decreased by 8.5% with stubble mulching. ST also increased wheat WUE by 5.8% with no mulching. Winter wheat WUE increased with conservation tillage during fallow period in fine and coarse soils, but decreased in medium soils (Figure 3E). The RR of wheat WUE to conservation tillage compared to CT was negative when MAP was 400–600 mm, but not significant when MAP was over the range. Wheat WUE RR was also negative when MAT was < 8 and > 15°C but negative when MAT 8–15°C.

### Mulching Effect on Precipitation Storage Efficiency and Soil Water Storage at Wheat Planting

**FIGURE 4:**
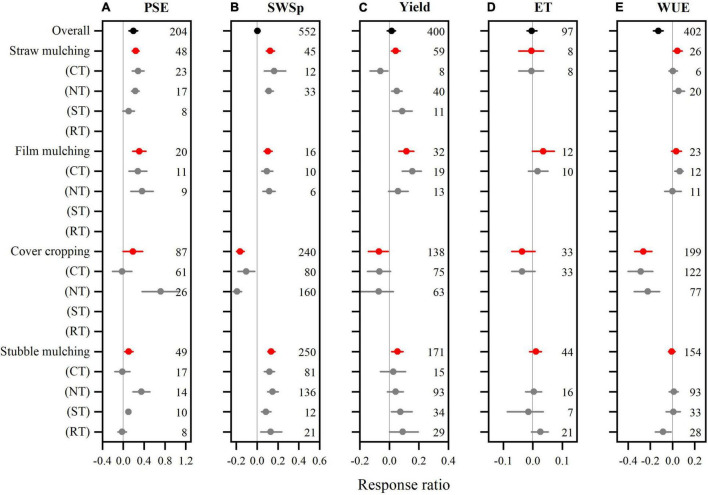
The mean response ratios of precipitation storage efficiency (PSE, **A**), soil water storage at wheat planting (SWSp, **B**), winter wheat grain yield **(C)**, evapotranspiration (ET, **D**), and water use efficiency (WUE, **E**) to fallow mulching practices compared to no mulching and their interactions with tillage methods. The horizontal line represents the bootstrapped 95% confidence interval. Conservation tillage methods are no tillage (NT), reduced tillage (RT), subsoil tillage (ST). The reference line (RR = 0) specifies no variation between conservation tillage and conventional tillage. Numbers accompanying the bootstrapped 95% confidence intervals designate the number of observations for comparisons.

**FIGURE 5:**
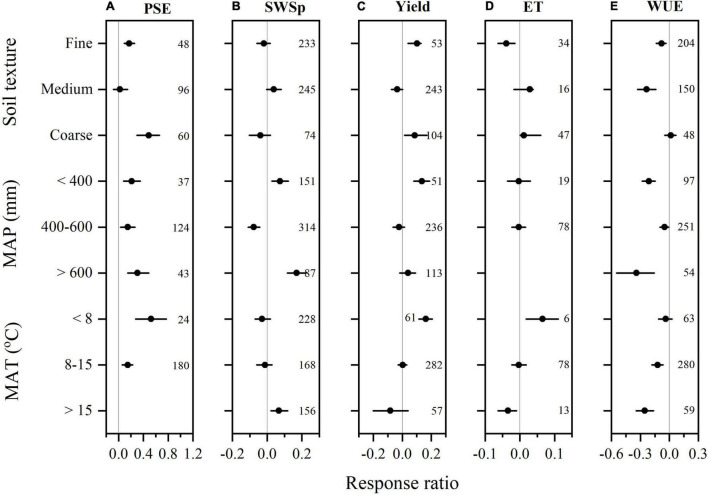
The mean response ratios of precipitation storage efficiency (PSE, **A**), soil water storage at wheat planting (SWSp, **B**), winter wheat grain yield **(C)**, evapotranspiration (ET, **D**), and water use efficiency (WUE, **E**) to fallow mulching practices compared to no mulching and their bootstrapped 95% confidence intervals (Horizontal line) as affect by soil texture, mean annual precipitation (MAP), and mean annual air temperature (MAT). The reference line (RR = 0) specifies no variation between conservation tillage and conventional tillage. Numbers accompanying the bootstrapped 95% confidence intervals designate the number of observations for comparisons.

We collected 204 and 552 paired observations for PSE and SWSp with fallow mulching practices (Figures 4A,B), respectively. High heterogeneities were found for the data as indicated by the high *Qt* values (211 and 516 for PSE and SWSp, respectively). Compared to no mulching, fallow mulching practices overall increased PSE by 19.4% (*P* < 0.05) compared to no mulching (Figure 4A). The categorical meta-analysis showed that all fallow mulching practices exhibited a positive effect on PSE, with increases of 23.9, 30.8, 19.1, and 10.8% (*P* < 0.05) for straw mulching, film mulching, cover cropping, and stubble mulching, respectively. Strong interactions with tillage were found for all fallow mulching practices except for film mulching. Cover cropping effect on PSE compared to no mulching was neutral with CT but positive with NT. Similarly, compared to no mulching, fallow stubble mulching had no effect on PSE with CT and RT, but increased PSE with NT and ST. Fallow mulching had no effect on PSE in medium soils, but increased PSE in fine and coarse soils with a greater RR value in coarse than fine soils (Figure 5A). The RR of PSE to fallow mulching compared to no mulching was not different with MAP and greater when MAT was <8°C than 8–15°C.

Not like PSE, the overall effect of fallow mulching practices on SWSp was not significant compared to no mulching (Figure 4B). Straw mulching, film mulching, and stubble mulching increased SWSp by 12.6, 10.2, and 13.4% over no mulching fallow (*P* < 0.05), respectively. However, cover cropping decreased SWSp by 16.3% (*P* < 0.05) compared to no mulching during fallow period. Effects of fallow mulching practices on SWSp were not interacted with tillage methods, and no significant differences were found in the RRs of SWSp among tillage methods for each mulching practice. The RRs of SWSp to fallow mulching were not significant in soil textures (Figure 5B). Fallow mulching increased SWSp when MAP was < 400 and > 600 mm but decreased when it was 400–600 mm. Positive effect of fallow mulching on SWSp was only observed when MAT was > 15°C.

### Mulching Effect on Winter Wheat Yield, Evapotranspiration, and Water Use Efficiency

The mulching responses were variable on 400 observations for wheat yield, 97 for ET, and 402 for WUE (Figures 4C–E). Data exhibited high heterogeneities with *Qt* values of 388, 98, and 406 for wheat yield, ET, and WUE, respectively. The mulching practices showed negative to positive RR on wheat yield, exhibiting an overall non-significant effect (Figure 4C). Compared to no mulching during fallow, straw mulching, film mulching, and stubble mulching increased winter wheat yield by 4.2, 6.2, and 5.5% (*P* < 0.05), respectively, while cover cropping decreased wheat yield by 7.0% (*P* < 0.05). Strong interaction with tillage methods was found for straw mulching. Straw mulching significantly decreased winter wheat yield with CT, but increased with NT and ST. Fallow mulching also decreased wheat yield in medium soil, but increased in fine and coarse soils (Figure 5C). The RR of wheat yield to fallow mulching compared to no mulching was only positive when MAP was < 400 mm and MAT < 8°C but not significant for other climatic conditions.

Fallow mulching overall had a neutral effect on ET compared to no mulching (Figure 4D). This is true for all mulching practices. Also, no interactions were found between mulching practices with tillage methods. Fallow mulching decreased ET in fine soil, but increased in coarse soil (Figure 5D). The RR of ET to fallow mulching compared to no mulching did not vary with MAP, and was positive when MAT was < 8°C but negative when MAT was > 15°C.

Winter wheat WUE was overall 12.8% lower with mulching than no mulching during fallow period (Figure 4E). Straw mulching increased wheat WUE by 4.4%, while cover cropping decreased by 26.3% compared to no mulching. Both film mulching and stubble mulching had no effect on wheat WUE. When combined with tillage methods, however, film mulching increased WUE with NT, and stubble mulching decreased with RT. Fallow mulching also decreased wheat WUE in fine and medium soils but had a neutral effect in coarse soil (Figure 5E). The RR of wheat WUE to fallow mulching compared to no mulching was not significant with MAP, and negative when MAT was >8°C.

### Correlations of Winter Wheat Yield, Evapotranspiration, and Water Use Efficiency to Soil Water Storage at Planting

**FIGURE 6:**
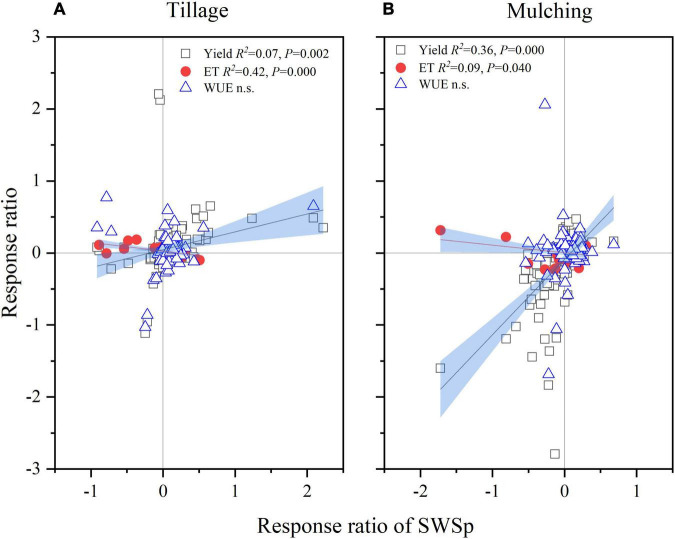
Correlations of the response ratio of winter wheat grain yield, evapotranspiration (ET), and water use efficiency (WUE) to that of soil water storage at wheat planting (SWSp) **(A,B)**.

The RR of winter wheat yield increased with increase of the RR of SWSp linearly for both tillage and mulching management practices (Figures 6A,B). About 7 and 36% increases in the RR of wheat yield can be explained by the increase of the RR of SWSp for tillage and mulching management practices, respectively. Similarly, The RR of ET was also related to with that of SWSp, and about 42 and 9% increases in the RR of ET can be explained by the increase of the RR of SWSp for tillage and mulching during fallow period. No correlation was found between the RR of wheat WUE and that of SWSp.

## Discussion

### Responses of Soil Water Storage to Fallow Tillage and Mulching Practices

Soil water storage during fallow is one of the critical factors affecting winter wheat yield in winter wheat monoculture system (Wang et al., 2018). Conservation tillage overall showed promises because of increased PSE and SWSp (Figures 2A,B), confirming our first hypothesis that conservation tillage methods improve soil water storage in fallow periods. The positive effects of conservation tillage on soil water conservation are probably due to the reduced soil disturbance, reduced soil bulk density, and improved aggregate stability by conservation tillage and mulching methods (Oyedele et al., 1999; Zhang et al., 2007). Previous studies also demonstrate water conservation benefits, although the relative effects on yield and water use vary with conservation tillage systems (Yan et al., 2005; Jin et al., 2007). However, the effect of conservation tillage during fallow period on soil water varied with tillage methods (Figures 2A,B). NT had a greater impact on PSE and SWSp than other conservation tillage practices during fallow, while RT had no impact on SWSp and ST significantly decreased SWSp compared to CT. Halvorson et al. (2000) and Jin et al. (2007) also reported that NT was the best tillage practice in fallow period for water conservation. During a 3-year study, soil water storage improved to varying degrees with different tillage practices irrespective of the volume of fallow period rainfall, but NT was more effective in fallow precipitation storage by increasing SWS and PSE compared to CT (Hou et al., 2012). Although some researchers reported that RT or ST after winter wheat harvest is useful to retain the rain water by increasing water infiltration and reducing runoff and evaporation (Jin et al., 2007; Hou et al., 2012), this meta-analysis suggests that they may not be efficient to conserve soil water during fallow period. We also found that the effect of NT on SWSp compared to CT interacted with mulching practices during fallow period. NT no mulching, NT straw mulching, and NT stubble mulching increased SWSp (Figure 2B), but NT cover cropping significantly decreased SWSp although it had a positive impact on PSE. This confirms the recent meta-analysis by Wang et al. (2021), who reported that SWSp of succeeding main crops reduced after cover cropping during fallow. Including cover crops during fallow period may consume more water for their establishment and growth (Ward et al., 2012; Wang et al., 2021).

Not like conservation tillage, mulching practice during fallow period overall had a neutral impact on SWSp despite its positive effect on PSE (Figures 4A,B). Such non-significant effect may not be conclusive since the positive effects of straw mulching, film mulching, and stubble mulching were neutralized by the negative impact of cover cropping, which is considered as a practice of biological mulching. Compared to no mulching, surface mulching with crop straw, stubble and plastic film can conserve soil water during fallow period through reducing soil moisture loss caused by evaporation (Fan et al., 2007; Gao et al., 2009), improving water penetration, saturated hydraulic conductivity, and soil water sorption (Blanco-Canqui and Lal, 2007), and lowering potential runoff (Jin et al., 2007). Cover cropping also tended to increase PSE due to increased soil water-related properties (Blanco-Canqui and Ruis, 2020), especially with NT. However, water loss through cover crop transpiration, combined with the potential water loss during the interval between cover crop termination and winter wheat planting, may lead to a reduction in SWSp with cover cropping (Figure 4B).

### Responses of Wheat Yield and Water Use to Fallow Tillage and Mulching Practices

Winter wheat grain yield overall increased with conservation tillage but remained non-significant with mulching practices during fallow period, which partly confirms our hypothesis. Hou et al. (2012) reported significant differences in wheat yield among conservation and conventional tillage systems with the grain yield increased by 9.6 and 10.7% with NT and ST compared to CT. Higher winter wheat yield with conservation tillage than CT was due to improved soil physical and chemical properties (Fabrizzi et al., 2005). Compared to CT, conservation tillage reduced soil disturbance, improved aggregate stability (Zhang et al., 2007) and water holding capacity (Hillel, 1998), which would be helpful to conserve soil water at wheat planting and correspondingly provide a buffer against short droughts during the growing season and increase wheat crop yield (Pikul and Aase, 1999; Pikul and Aase, 2003; Verhulst et al., 2011). Interestingly, conservation tillage requires less manpower and energy for agricultural production (Zhang et al., 2009), and provide long-term benefits such as improved soil structure, reduced farm traffic and soil erosion (Wang et al., 2008). The non-significant yield response to fallow mulching, however, might be a result of counteraction between cover cropping and other mulching practices as discussed above. Strong correlations between the RR of wheat yield and that of SWSp for both tillage and mulching practices (Figure 6), indicate that enhancement of soil water storage during fallow through proper fallow management can increase wheat yield. Similarly, Wang et al. (2021) also reported a significant correlation between the RR of succeeding crop yield and that of SWSp under cover cropping systems, which emphasizes the importance of SWSp for crop production. In improved SWS conditions, crops would consume more soil water stimulating their growth (Wang et al., 2004). Strong interaction between tillage and mulching was also found for wheat yield in this study. NT no mulching, NT straw mulching, RT no mulching and ST film mulching increased wheat yield compared to other combinations, indicating that residue cover or with reduced tillage with NT and RT can store more soil moisture by minimizing rainfall water loss and soil surface evaporation and eventually increase crop yield (Wang et al., 2011).

The overall effect of conservation tillage methods on ET was negligible (Figure 2D). This is in consistent with the finding by López and Arrúe (1997), who reported that the total ET was generally similar for all tillage treatments. A large proportion of ET lost as evaporation could explain this lack of differences, and decreased ET under conservation tillage during vegetative growth of crop, demonstrating the treatment’s inability to meet crop water needs in the rainfed cropping systems. The reduced ET with conservation tillage may relate to higher levels of soil strength (Schmidt and Belford, 1994) and/or insufficient amounts of crop residues on the soil surface (Gibson et al., 1992). Our meta-analysis also showed that fallow mulching overall did not affect ET but cover cropping tended to decrease it (Figure 4D). Similarly, He et al. (2016) reported that planting legume and straw-legume as cover crops during fallow decreased winter wheat ET. The reduced ET due to cover cropping could be explained by lower soil water storage at wheat planting, which could result in less accessible soil moisture supply, reducing water evaporation from the soil surface and limiting winter wheat transpiration (Zhang et al., 2007).

Wheat WUE was not affected by conservation tillage methods (Figure 2E) but decreased by fallow mulching practices (Figure 4E). Crop water use depends on both SWSp and the amount and distribution of precipitation in crop growing season (Hemmat and Eskandari, 2004, 2006; Huang et al., 2006). This study indicates that the water conservation during fallow period due to conservation tillage may not always lead to a better water use in cases of no more precipitation occurred during growing season. Although a linear relationship between wheat grain yield and WUE has been reported by several researches (Huang et al., 2005; Lenssen et al., 2014), our findings contradicted previous reports of higher WUE with mulching than without in rainfed agriculture systems (Deng et al., 2006; Chakraborty et al., 2010). However, straw mulching during fallow period exhibited a positive impact on wheat WUE, indicating that straw mulching could be a better choice for wheat water use compared to other mulching practices (Wang et al., 2018).

### Variances With Soil Textures and Climatic Conditions

The effects of tillage methods and mulching practices during fallow period on soil water conservation and wheat yield and water use also varied with soil textures and climatic conditions (Figures 3, 5). Soil texture generally affects the hydraulic properties of soil, which may affect the water loss with different fallow management practices (Hammel et al., 1981; Gajri et al., 2002). The positive impact of conservation tillage on SWSp and wheat yield and WUE in coarse soil was in accordance to previous finding by Carter and Tavernetti (1968), who revealed that crop yield response to conservation tillage was directly linked with soil strength in California (United States). Similarly, there is significant evidence that conservation tillage in coarse-textured soils creates a continuous low-strength slit for root expansion, providing interim relief to crops, and resulted in a significant increase in wheat yield (Gajri et al., 1991). Compared to no mulching, fallow mulching also increased PSE and wheat yield in fine and coarse-textured soils. The results support the findings of Gajri et al. (1994) who revealed that mulching increased grain yield in coarse-textured soils for all the 10 years studied. Similarly, Triplett et al. (1970) reported that mulching increased crop yield in coarse-textured soils, but decreased crop yield on the medium-textured soils. Gajri et al. (1994) found that mulching with conservation tillage increased grain yield in coarse-textured soils. Conservative tilled or mulched soils could help plants to scavenge water and nutrients from the subsoil more efficiently (Arora et al., 1991).

Climate variables might bring positive or negative influences on crop yield (Saddique et al., 2020). In Pacific Northwest (United States), most of the water loss occurs in fallow land in a dry layer of 10 cm or more in thickness, while large variations in diurnal temperature occur in the upper 15 cm of soil may affect the dry layer vapor flow (Papendick et al., 1973). The fallow duration under winter wheat monoculture generally ranges from 3 months (Wang et al., 2011) to 24 months (Huang et al., 2015). Using the data for MAP instead of the precipitation occurred during fallow period or growing season may have led to some bias in this meta-analysis study, as wheat production is affected by not only SWSp but also water supply during growing season (Brown and Rosenberg, 1997; Wang et al., 2011; Maitah et al., 2021). In Eastern United States, where the relationship between crop yield and precipitation revealed that moisture scarcity, however, herded crop yield (Huang et al., 2015). Fallow mulching can maintain an even soil temperature, increase soil moisture (Barman et al., 2008), and reduce soil evaporation (Liu et al., 2002). Mean annual temperature (MAT) significantly affected PSE and wheat yield, and maximum PSE and yield were obtained at 8–15°C with tillage (Figure 3), and at < 8°C with mulching (Figure 5), which can be explained by the crop yields normally decrease with increasing temperature because of the shorter phenological phases (Brown and Rosenberg, 1997).

## Conclusion

This meta-analysis summarized the effects of conservation tillage methods and mulching practices during fallow period on soil water storage, crop yield and water use under winter wheat mono-cropping systems and demonstrated NT with straw mulching as the most suitable practice for soil water conservation and dryland crop production. Conservation tillage during fallow period overall increased PSE, SWSp and wheat yield but did not affect ET and WUE. NT is more efficient to conserve soil water during fallow period compared to RT and ST. Fallow mulching practices overall increased PSE but had a non-significant impact on SWSp, wheat yield, and ET. Compared to straw mulching, film mulching, and stubble mulching during fallow period, cover cropping as a biological mulching decreased SWSp, wheat yield, and WUE significantly. Wheat WUE was significantly enhanced by fallow straw mulching. The effects of tillage method and mulching practices were strongly interacted for soil water conservation and wheat yield and water use, and varied with soil textures and climatic conditions. NT in combination with straw mulching significantly increased SWSp, PSE, wheat yield, and WUE and can be the best fallow management practice for winter wheat production in varying edaphic and climatic conditions.

## Data Availability Statement

The original contributions presented in the study are included in the article/supplementary material, further inquiries can be directed to the corresponding author/s.

## Author Contributions

MA, SZ, JW, and AS conceived the review, drafted, and finalized the manuscript. MT and SF helped to improve the draft by providing useful suggestions and information. All authors contributed to the article and approved the submitted version.

## Conflict of Interest

The authors declare that the research was conducted in the absence of any commercial or financial relationships that could be construed as a potential conflict of interest.

## Publisher’s Note

All claims expressed in this article are solely those of the authors and do not necessarily represent those of their affiliated organizations, or those of the publisher, the editors and the reviewers. Any product that may be evaluated in this article, or claim that may be made by its manufacturer, is not guaranteed or endorsed by the publisher.

## Footnotes

1
https://scholar.google.com/

2
https://www.webofknowledge.com/

3
http://getdata-graph-digitizer.com/index.php

4
www.whatsmygps.com
